# Microbiome metabolic capacity is buffered against phylotype losses by functional redundancy

**DOI:** 10.1128/aem.02368-24

**Published:** 2025-01-30

**Authors:** Kayla Cross, Noelle Beckman, Benjamin Jahnes, Zakee L. Sabree

**Affiliations:** 1 Department of Microbiology, Ohio State University 215854 , Columbus, Ohio, USA; 2 Utah State University 4606 , Logan, Utah, USA; 3 Department of Evolution, Ecology and Organismal Biology, Ohio State University 170262 , Columbus, Ohio, USA; Norwegian University of Life Sciences, Ås, Norway

**Keywords:** microbial ecology, cockroach, 16S RNA, functional capacity, insect model system

## Abstract

Many animals contain a species-rich and diverse gut microbiota that likely contributes to several host-supportive services that include diet processing and nutrient provisioning. Loss of microbiome taxa and their associated metabolic functions as result of perturbations may result in loss of microbiome-level services and reduction of metabolic capacity. If metabolic functions are shared by multiple taxa (i.e., functional redundancy), including deeply divergent lineages, then the impact of taxon/function losses may be dampened. We examined to what degree alterations in phylotype diversity impact microbiome-level metabolic capacity. Feeding two nutritionally imbalanced diets to omnivorous *Periplaneta americana* over 8 weeks reduced the diversity of their phylotype-rich gut microbiomes by ~25% based on 16S rRNA gene amplicon sequencing, yet PICRUSt2-inferred metabolic pathway richness was largely unaffected due to their being polyphyletic. We concluded that the nonlinearity between taxon and metabolic functional losses is due to microbiome members sharing many well-characterized metabolic functions, with lineages remaining after perturbation potentially being capable of preventing microbiome “service outages” due to functional redundancy.

**IMPORTANCE:**

Diet can affect gut microbiome taxonomic composition and diversity, but its impacts on community-level functional capabilities are less clear. Host health and fitness are increasingly being linked to microbiome composition and further modeling of the relationship between microbiome taxonomic and metabolic functional capability is needed to inform these linkages. Invertebrate animal models like the omnivorous American cockroach are ideal for this inquiry because they are amenable to various diets and provide high replicates per treatment at low costs and thus enabling rigorous statistical analyses and hypothesis testing. Microbiome taxonomic composition is diet-labile and diversity was reduced after feeding on unbalanced diets (i.e., post-treatment), but the predicted functional capacities of the post-treatment microbiomes were less affected likely due to the resilience of several abundant taxa surviving the perturbation as well as many metabolic functions being shared by several taxa. These results suggest that both taxonomic and functional profiles should be considered when attempting to infer how perturbations are altering gut microbiome services and possible host outcomes.

## INTRODUCTION

Animal gut microbiomes are often complex environments due to their being comprised of a few (e.g., honeybees: ~10 species) to many (e.g., mammals: >300 species) (1) bacterial species. Each lineage bears an array of distinct molecular constituents and is involved in pairwise and higher-order interactions (2). 16S rDNA amplicon sequencing is the most common approach for describing microbiome compositionality by enumerating representative amplicons that can also reveal a taxonomic dimension of the microbiome (3). It is increasingly common to infer microbiome functional capacity from amplicon-based microbiome composition using reference genome content from taxonomically proximal lineages with the assumption that detected members of the microbiome will also bear those characteristics (4). Combining both taxonomic and functional dimensions of microbiomes enables researchers to develop testable hypotheses about ecosystem-level (5) functions, how various perturbations (e.g., changes in host diet, onset of host disease, and host aging) impact them, and potentially link specific lineages to these host-relevant functions (6).

Comparative analysis of gut bacterial communities has previously revealed variable taxonomic, but stable functional compositions across the same or related host species, and similar environmental niches (7, 8). Many ecologically relevant bacterial metabolic functions are polyphyletic or conserved across distantly related bacterial species, including metabolite respiration and nutrient acquisition and uptake pathways (7, 9). Reductions in microbiome taxonomic alpha-diversity are often interpreted as consequential alterations of microbiome ecological trait richness (9, 10) that lead to loss of functions and, to appropriate a familiar telecommunications phrase, microbiome “service outages.” Maintenance of metabolic functions through community perturbations can occur by taxon resilience or functional redundancy. The former simply describes when lineages survive perturbations and neither they nor their metabolic services are lost (11). Functional redundancy is when metabolic services are shared by multiple lineages comprising the community (11) and they remain in the community via lineages that survive after a taxon diversity loss event. Metabolic pathways that are redundant within a community can exist as *identical pathways* found in distinct taxa or *distinct pathways* that yield identical outcomes in distinct taxa. We sought to examine to what degree shifts in taxonomic diversity and metabolic functional capacity are decoupled in a complex gut microbiome following a perturbation.

Dietary changes can alter gut microbial community composition and diversity (12, 13) and thus present a useful model for examining the taxonomic-functional microbiome compositional relationship. Given a sufficiently taxonomically rich microbiome, we expect that diet-induced alterations in taxonomic composition will have a minimal effect on community-level metabolic pathway composition. The American cockroach (*Periplaneta americana*) is an ideal experimental model because it has a species-rich gut microbiome (~300 taxa) spanning 19 phyla, with members of the Bacteroidota, Bacillota, and Pseudomonadota predominating (14), easily reared and it is amenable to consume a wide range of diets. We expected that feeding *P. americana* nutrient-imbalanced diets over several weeks and characterizing their gut microbiomes would facilitate examining the nonlinearity between taxon composition and predicted metabolic capacity. We observed that inferring microbiome ecosystem functional changes based on taxonomic censuses alone does not account for how community-level microbiome functional capacity is maintained through metabolic functional polyphyly. As such functional redundancy likely buffers against dramatic species declines in complex microbiomes.

## MATERIALS AND METHODS

### Sample preparation

Female *P. americana* insects reared to adults on nutritionally balanced dog food in a lab colony were used in these studies. Two treatment groups were comprised of seven individuals each who were fed either protein-enriched or cellulose-enriched diets for up to 8 weeks. A third population also comprised of seven individuals was maintained on nutritionally balanced dog food as a positive control. All insects were housed individually, and frass (insect feces) was collected from all individuals each week. Insect deaths were observed in both unbalanced diet treatments, reducing the number of total samples we could collect for these treatments (Table S1). Insect deaths in the unbalanced diet treatments likely resulted from nutrient deprivation as no insects fed the balanced diet died during the 8-week treatment period. Frass samples from each individual insect were collected within 48 h of deposition and stored in RNA Later (ThermoFisher Scientific) at −80°C until DNA extraction using the MoBio PowerSoil kit (Qiagen) according to the manufacturer’s instructions; no frass samples were pooled. DNA concentration was determined using a Qubit UV spectrophotometer. Frass sample DNA extracts that yielded 1.5 kb amplicons in diagnostic PCR amplifications using universal and bacteria-specific primers (27F and 1492R) (15) were deemed acceptable for microbiome analyses and selected for Illumina MiSeq-based 16S rDNA amplicon sequencing. Frass DNA extracts were sent to the Argonne National Lab for V4 amplicon generation and 2 × 251 bp paired-end sequencing using v2 chemistry and 515F and 806R primers (16).

All sequence reads (17) were assembled and quality-trimmed to 251 nt fragments. Samples with fewer than 5,000 reads (*n* = 49) in first-pass ecological analyses were excluded, resulting in 117 samples being used in final analyses (Table S1). Fewer than four samples per week for weeks 5 through 8 for insects fed cellulose-enriched diets passed quality filtering because of high amounts of erroneous reads. As such, data from this treatment for these weeks were not included in our final community analyses. 3,125 operational taxonomic units (OTUs) were generated at the >95% sequence identity in uclust (v1.2.22) (18). OTUs were taxonomically assigned, and hereafter referred to as “phylotypes” (19), by nucleotide BLAST searches of Arb-SILVA “SSU” database (version 126) (20, 21). Phylotypes represented by ≤10 amplicons in ≤10 samples each were excluded from community analysis as they were indistinguishable from sequencing artifacts. All OTUs were assigned to 505 bacterial family-level phylotypes and 144 remained post-filtering.

### Quantification of bacterial abundance by qPCR

Bacteria present within frass samples were quantified by performing absolute quantification qPCR using frass sample DNA extracts to generate amplicons with primers (Uni331F, TCCTACGGGAGGCAGCAGT and Uni797R, GGACTACCAGGGTATCTATCCTGTT; [22]) targeting an internal region of the 16S rDNA gene. Primer efficiency was ≥90%. 466 bp 16S rRNA gene amplicons were generated from a single frass sample, and these were cloned into the pGEM-T Easy vector (Promega) and transformed into JM109 Competent Cells as described in the manufacturer’s protocol.

Plasmid containing the 466 bp amplicon was extracted from a 5 mL overnight culture of a single white colony using a QIAGEN plasmid miniprep kit. Purified plasmid DNA was used to generate a standard curve across six dilutions with three technical replicates per dilution. Frass samples were run with three technical replicates per biological sample. Standard curve and frass samples were run simultaneously on the same plate. Balanced and protein-enriched diets had three biological replicates for weeks 1, 4, and 8, while cellulose-enriched had three biological replicates for week 1 and one biological replicate for week 4. Negative controls included a set of triplicate reactions without DNA or without the primers. For all qPCR reactions, Luna Universal qPCR Master Mix by New England Biolabs was used, and thermocycler conditions with a melt curve analysis were programmed as recommended.

### Community ecology analysis

The impact of unbalanced diets on phylotype community structure was examined using multivariate analysis of Sørenson (i.e., phylotype presence-absence) and Bray-Curtis (i.e., phylotype relative abundance) pairwise dissimilarity measures (23). Nonmetric multidimensional scaling (NMDS) was used to visualize results (100 random starts, function “metaMDS” in Vegan [24]). Two additional samples were flagged as outliers by visual identification in NMDS plots and these were excluded from further analysis. A PERMANOVA test was also used to assess the differences in phylotype community composition between unbalanced and balanced diet treatments (999 permutations, function “adonis2” in Vegan). Due to repeated measures and non-independence of samples, the permutations were restricted within individuals (25). PERMANOVA is sensitive to differences in multivariate dispersion and significant differences among groups could be due to differences in centroids, differences in dispersion, or both. Multivariate homogeneity of group dispersions was analyzed using a permutation test (9,999 permutations; functions “betadisper” and “permutest” in vegan). Multivariate group dispersions across weeks were not significantly different (*P* < 0.05), and therefore those results were not included (Bray-Curtis: *F*_7, 115_ = 0.3659, *P* = 0.924, Sørenson: *F*_7, 115_ = 1.8342, *P* = 0.089). Pairwise comparisons were conducted to assess which differences in diet type were responsible for significant shifts in multivariate dispersion, and *P* values were adjusted using the Benjamini-Hochberg False Discovery Rate (FDR) (26).

For alpha-diversity, we measured phylotype richness, Hill-Shannon Diversity, and Hill-Simpson Diversity. These are special cases of Hill diversity that differ only in how they scale rarity and are algebraically related to commonly used diversity indices (27–29). Phylotype richness (the number of phylotypes) equally counts all phylotypes and thus emphasizes and is sensitive to rare phylotypes. Hill-Simpson Diversity emphasizes, and is most sensitive to, common phylotypes. Hill-Shannon Diversity falls between the two and emphasizes neither common nor rare phylotypes (30). We used linear mixed models (LMMs) to analyze the influence of diets on repeated measures of gut phylodiversity over time. The number of weeks, type of diet, and their interaction were included as fixed effects, and individual was included as a random intercept to account for correlation among repeated measures. LMMs were fit using restricted maximum likelihood estimation. Due to the uncertainty in calculating degrees of freedom (31), 95% confidence intervals of parameter estimates were obtained using bootstrapping (1,000 simulations with the percentile method) (32) and estimates were considered to be significantly different from zero if the confidence intervals do not overlap zero. The iNEXT (33) R package (version 4.0.2 [34]) was used to calculate phylotype richness, Hill-Shannon Diversity, and Hill-Simpson Diversity over time for taxonomic composition analysis and LMMs were fit using “lme4” in R (35). All analyses were performed in R version 4.2.2.

### Prediction of functional pathways

Phylogenetic Investigation of Communities by Reconstruction of Unobserved States (PICRUSt2) (4) was used for metagenomic functional profile prediction of bacteria present in the gut communities across different diet types. OTU sequences and abundance table of bacterial taxa were inputted into the PICRUSt2 pipeline. Sequences were aligned to a reference tree based on >20,000 16S sequences from the Integrated Microbial Genomes database (36) using EPA-NG (37) and gappa (38). Sixteen out of 3,125 (5%) OTUs were removed due to the nearest-sequenced taxon index (NSTI) value being greater than 2.0 per the PICRUSt2 recommendations. 95% of *P. americana* OTU mapped to a reference genome. The NSTI value for each OTU represents the branch length between each OTU and the closest reference sequence. PICRUSt2 uses NSTI values as a guide for how similar each OTU is to an existing reference sequence. Gene family numbers inferred with castor (39) were multiplied by the abundance table, yielding enzyme classification (EC) per OTU for all samples. ECs are matched to pathways and pathway abundances were calculated using MinPath (40) for MetaCyc functional pathway profiles.

### Measuring metabolic capacity

We used PICRUSt2 to examine the perturbation-induced dynamics of *metabolic capacity*, which is defined as the microbial gene content of a community capable of encoding all possible metabolic processes therein. By default, PICRUSt2 uses metabolic enzyme encoding genes from all genomes represented by OTUs to construct pathways because it assumes cross-feeding interactions between microbiome constituents, and it does not require that a single genome associated with each OTU-associated taxon have all genes needed to independently produce a pathway. PICRUSt2 was run using this default to identify “total pathways.” To identify and enumerate complete metabolic pathways that could be associated with a single genome, PICRUSt2 was run separately with the “--per_sequence_contrib” command to yield the “per-taxon” pathways (4; https://github.com/picrust/picrust2). Pathways in the “total pathways” group but not in the “per-taxon” group were assigned to the “obligately-shared” pathways group, which was defined as those pathways that required enzymes encoded by genes present on two or more genomes from two or more taxa to be complete pathways. Metacyc (41) was used to identify “variant” pathways whose constituent enzymes were distinct, but the pathways yielded the same metabolites. As such, these genes can come from the genomes of two or more lineages. However, the default assumes that all genes are shared across OTUs through cross-feeding interactions and does not mean that each OTU has enough genes to independently produce that pathway. A pathway was deemed present if it was detected in at least 25% of samples per treatment for weeks examined.

The effect of unbalanced diet treatments on microbiome metabolic capacity was observed by comparing the number of inferred pathways present in microbiome samples between balanced and unbalanced diet treatments. Microbiome samples from age-matched insects were used in these comparisons: weeks 3 and 4 of balanced diet (*n* = 13) versus weeks 3 and 4 of cellulose-enriched diet (*n* = 10); and weeks 7 and 8 of balanced diet (*n* = 14) versus weeks 7 and 8 of protein-enriched diet (*n* = 10).

## RESULTS

### Microbiota composition varied with diet

**Fig 1:**
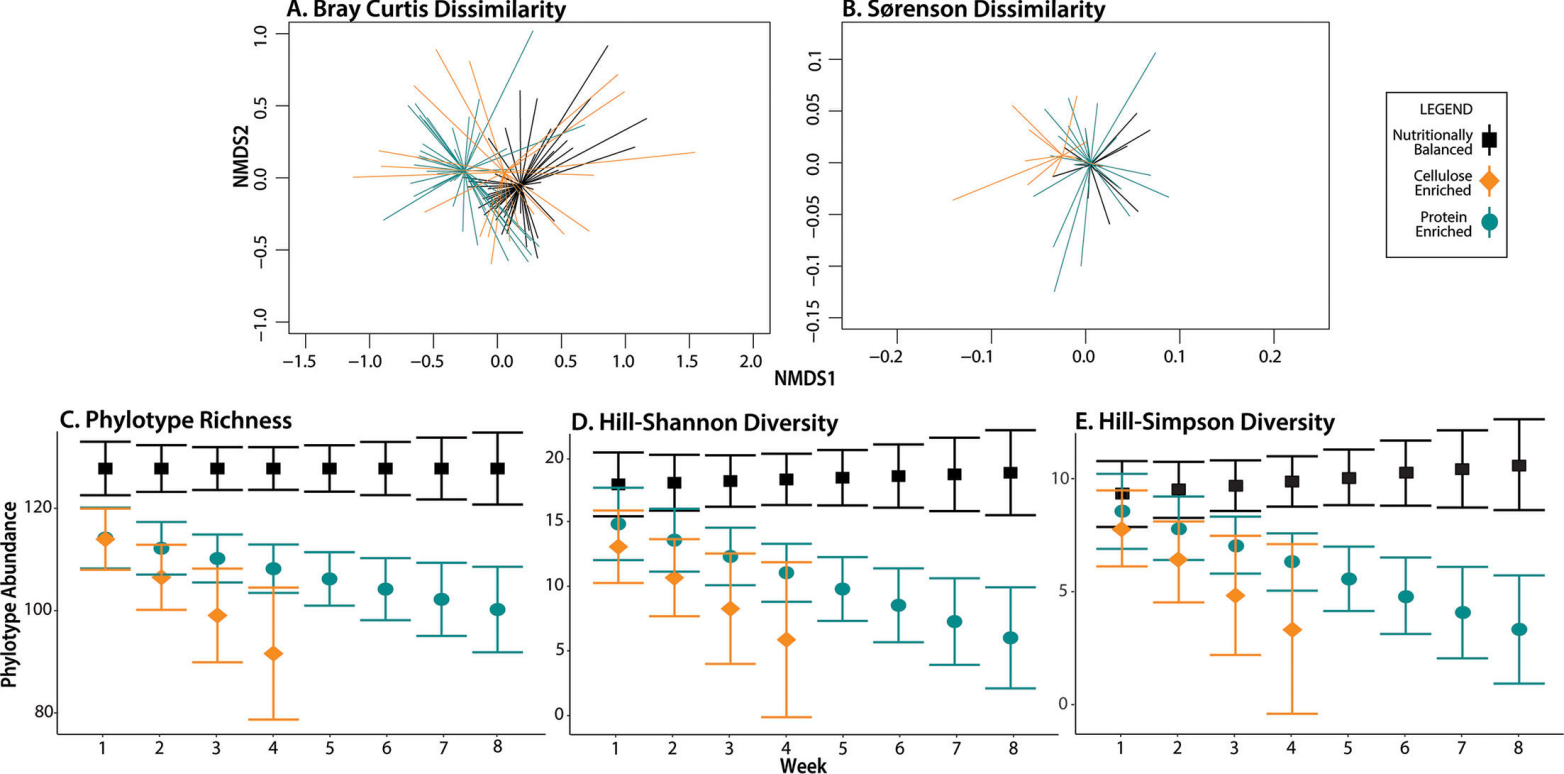
Unbalanced diets reduce alpha-diversity and define frass microbiome. Frass microbial community structure from insects fed unbalanced diets differs as indicated by differing centroids and dispersion for communities based on both relative abundance (Bray-Curtis dissimilarity; **A**) and presence-absence (Sørenson dissimilarity; **B**) of phylotypes. Unbalanced diet-induced changes in alpha-diversity were observed using phylotype richness (**C**), Hill-Shannon diversity (**D**), and Hill-Simpson diversity (**E**) metrics. Error bars represent two standard deviations from the mean.

**Fig 2:**
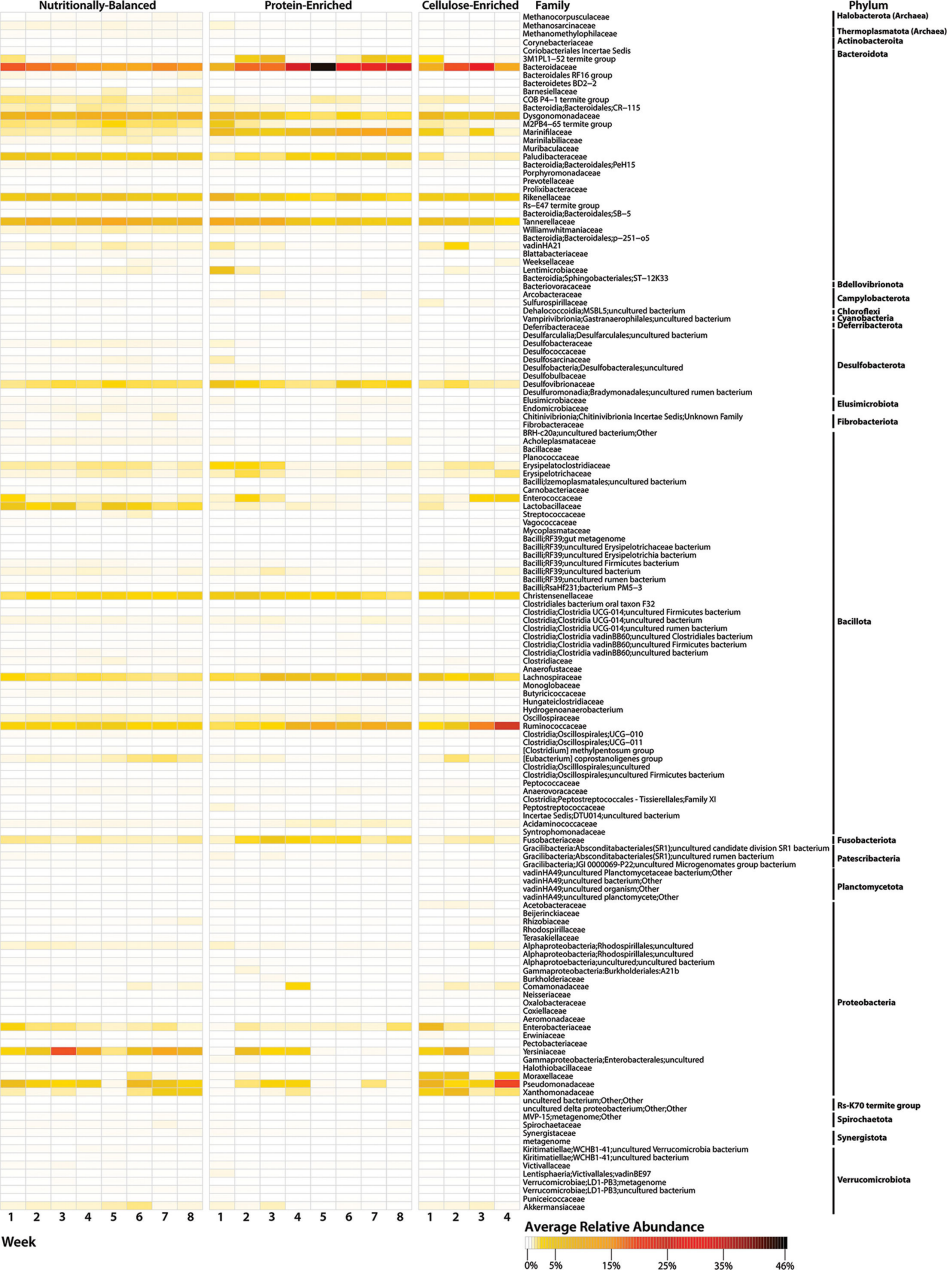
Relative abundances of *P. americana* fecal microbiome phylotypes as altered by diet. Relative abundances of family-level phylotypes averaged across all samples per week for each treatment are shown. Archaeal and bacterial families are organized by taxonomic hierarchy. White cells represent a relative abundance of 0%. Full taxonomic hierarchy names are included for any bacterial families classified as “other” and “unknown.”

Diets defined microbiomes in NMDS plots as observed in treatment-defined centroid translocation (Fig. 1A and B) and PERMANOVA analyses (Bray-Curtis index: *dF* = 2, *P* = 0.0002; Sorensen index: *dF* = 2, *P* = 0.0181; Table S2). Although protein-enriched and balanced diets had similar microbiome composition as indicated by overlapping centroids (Fig. 1B), distinct sample dispersion patterns were observed, and pairwise comparisons confirmed differences between balanced diet versus protein-enriched diet or balanced diet versus cellulose-enriched diet treatments (Table S3). Insects fed unbalanced diets had their family-level alpha-diversity reduced by 21–28% of the alpha-diversity observed in balanced diet fed insects (Fig. 1C through E, Table S4). In total, 144 family-level phylotypes spanning 21 phyla were identified across all samples for all diets, with ~70% being <1% of the average relative abundance (Fig. 2), and Bacteroidaceae, Dysgonomonadaceae, Paludibacteraceae, Rikenellaceae, Tannerellaceae, Lactobacillaceae, Christenellaceae, Pseudomonadaceae, Fusobacteriaceae, Yersiniaceae, and Ruminococaceae were among the top 15 most abundant phylotypes in all treatments (Fig. 2).

### Diet had a minimal effect on microbiome metabolic capacity

Average taxon relative abundances appeared to vary between balanced and unbalanced diet treatments, but average metabolic pathway relative abundances appeared less turbulent (Fig. S2), which suggested that metabolic capacity was not as affected by observed taxon alpha-diversity declines. Metabolic capacity describes all complete metabolic pathways encoded by phylotypes comprising a microbiome, and we identified 369 out of 370 total pathways (Table S5) that were present in the balanced diet-treatment microbiome (Table S5). One additional pathway was detected in <25% of all balanced diet microbiome samples. At the end of the treatment periods, protein-enriched diet treatment had 367 total pathways while cellulose-enriched diet treatment had 370 total pathways. Of the total pathways, 32 “obligately-shared” pathways were identified, of which 30 were detected in ≥75% of all samples from weeks 1 and 2 of balanced diet treatments (Table S6). Prevalence of these pathways in samples from weeks 7 and 8 for balanced and protein-enriched diet treatments were compared. All 32 were prevalent in the balanced diet treatments, and 30 were prevalent in protein-enriched diet treatments (Table S6). Prevalence of “obligately-shared” pathways in samples from weeks 3 and 4 for balanced and cellulose-enriched diet treatments were compared. Twenty-six were prevalent in the balanced diet treatments, and 31 were prevalent in cellulose-enriched diet treatments (Table S6). “Per-taxon” pathways represented 338 of the 370 total pathways, and 337 of the 338 of these pathways were prevalent in balanced diet treatment samples in weeks 1 and 2, 333 of the 338 at weeks 3 and 4, and 338 of the 338 at weeks 7 and 8 (Table S7). 337 of the 338 “per-taxon” pathways were prevalent in protein-enriched diet treatment samples at weeks 7 and 8 as well as in cellulose-enriched diet treatments at weeks 3 and 4 (Table S7).

**Fig 3:**
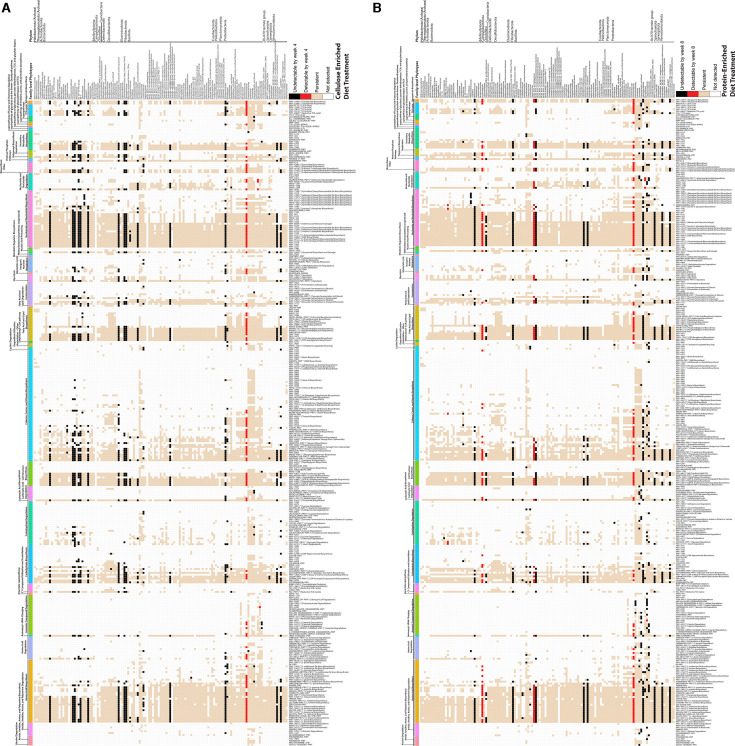
Phylotype loss has minimal impact on metabolic capacity due to pathway redundancy across phylotypes. Pathway presence (non-zero values) and absence (zero values) in cellulose-enrichment (**A**) and protein-enrichment (**B**) diet treatments were compared to balanced diet treatment. Pathways remained detectable across the treatment weeks (tan), were undetectable across treatment weeks (white), detectable only at the end of the treatment (red), or undetectable at the end of the treatment (black). Comparisons for metabolic pathway detectability were between microbiome samples of age-matched insects (i.e., balanced diet weeks 3 and 4 vs cellulose-enriched diet weeks 3 and 4; balanced diet weeks 7 and 8 vs protein-enriched diet weeks 7 and 8). The *y*-axis depicts bacterial and archaeal families ordered by phylum. The *x*-axis is MetaCyc pathway names grouped by a broad MetaCyc category (top) and ordered within the category from most shared to least shared pathways (bottom). Pathways that are a member of a variant class by MetaCyc are indicated by asterisks, followed by the variant class name. Higher resolution versions of these figures can be found in the supplemental material as Fig. S3A and S3B.

While 8% of the “per-taxon” pathways were associated with no more than one family-level phylotype, the large majority of “per-taxon” pathways were found in two or more phylotypes (Fig. 3A and B; Fig. S3A and B). When individual “per-taxon” pathways associated with specific bacterial families were observed to not be detected at the end of either unbalanced diet treatment (Fig. 3A and B, black boxes; Fig. S3A and B), these pathways were always detected in other bacterial families that remained (Fig. 3A and B, beige boxes; Fig. S3A and B). Furthermore, many functionally orthologous “variant” pathways were observed, including those “per-taxon” pathways found only in one bacterial family (Fig. 3A and B, asterisks on pathway labels; Fig. S3A and B). Finally, we performed quantitative PCR to determine if unbalanced diets reduced gut bacterial density, which might indicate that the microbiome could not use the provided nutrients even if the inferred metabolic capacity suggested otherwise. Bacterial cellular abundance significantly varied both within and between treatments throughout the experiment; however, there was not a reduction in the quantity of bacteria due to an unbalanced diet compared to individuals fed a balanced diet during week one (Fig. S1, Wilcoxon significance test, *P* < 0.05).

## DISCUSSION

This investigation sought to examine how complex microbiomes have built-in functional redundancies that enable them to continue to provide community-level services despite changes in phylotype diversity resulting from environmental disturbances. Diet-based microbiome perturbations of the microbial species-rich American cockroach gut microbiome were used to model the nonlinear relationship between microbiome taxonomic alpha-diversity and metabolic capacity. Nutrient-enriched diets were used to alter the gut microbiome taxonomic composition as this has been an effective approach in other animals with species-rich microbiomes (42–44). Up to 30% of family-level phylotypes comprising the microbiome were below detection following unbalanced diet treatments. In a related study, the relative abundances of phylum-level taxa were observed to remain stable in American cockroach gut microbiomes after hosts were fed several nutritionally distinct diets for 2 weeks (14), but family-level taxon diversity dynamics were not reported and were likely masked at the phylum level. Unbalanced diet-linked reductions of family-level taxon diversity were observed in many phyla described in reference (14), including prevalent phyla, like Bacteroidota, Bacillota, and Pseudomonadota (Fig. 2). Our data suggest that diet-driven gut bacterial taxonomic dynamics in *P. americana* are better resolved at the family- or genus-level rather than at the phylum-level.

While up to 30% of bacterial families were lost following feeding on unbalanced diet treatments, the remaining microbiome taxa following either treatment were capable of encoding >99% of the total metabolic pathways observed in positive control microbiomes (Fig. 3A and B, beige boxes; Fig. S3A and B). The observed nonlinearity between taxonomic alpha-diversity reductions and stable metabolic capacity was due to many functions being shared by more than one phylotype.

Despite considerable functional redundancy, the prevalence of a few metabolic pathways was diminished following unbalanced diet treatments. The aerobactin synthesis pathway (AEROBACTINSYN-PWY) is responsible for the iron-binding siderophore aerobactin biosynthesis, and it was uniquely associated with members of the Enterobacteriaceae in this study. While this pathway was prevalent at the initiation of the treatment period, it was no longer prevalent in the microbiomes of insects fed a cellulose-enriched diet (Fig. 3A; Table S7; Fig. S3A and B). Biological production of methane (METHANOGENESIS-PWY) and production of coenzyme B (P241-PWY) were uniquely performed by members of the Methanocorpusculaceae in this study and both unbalanced diets negatively affected the prevalence of this function (Fig. 3A and B; Table S7; Fig. S3A and B). Neither of these bacterial families was significantly diminished as a result of the unbalanced diet feedings, suggesting that these metabolic functions may be encoded in the genomes of some but not all genera or species within these families. Further experimental work is needed to determine what are possible ecological implications of the reductions of these metabolic functions. It was also observed that the numbers of prevalent (i.e., in >25% of treatment-defined samples per week) metabolic pathways in the balanced diet treatment microbiomes varied by ±1–10%, relative to weeks 1 + 2, over the 8-week period in the “obligately-shared” and “per-taxon” metabolic pathway groups (Table S6 and S7). While this could be due to experimental limitations (i.e., taxa being temporarily below the limit of detection), it is worth acknowledging that sequencing-based microbiome content analyses are snapshots of highly dynamic systems.

Nearly all described metabolic pathways were resilient to taxonomic diversity loss because (i) multiple taxa each have the same complete metabolic pathway (e.g., “per-taxon” pathways), (ii) multiple taxa were contributing to complete a single metabolic pathway (e.g., “obligately-shared” pathways), and/or (iii) the presence of functionally orthologous “variant” pathways that use distinct enzymatic repertoires to yield identical metabolites produced by other pathways. These features describe the multiple layers of functional redundancy within a phylotype-rich microbiome that can potentially buffer microbiome metabolic capacity against perturbations.

A useful layer of information missing from this analysis is metabolic pathway expression patterns for detectable phylotypes, and orthologous gene expression is potentially regulated by interspecies interactions within the gut. Up to 78% of orthologous genes have been observed to be downregulated when taxa coexist with a taxon already expressing that gene (45). For example, starch phosphorylase expression will be silenced in *Catenibacterium mitsuokai* (phylum Bacillota) when *C. mitsuokai* is coexisting with *Bacteroides caccae* (phylum Bacteroidota) (45). Metabolomics, metaproteomics and metatranscriptomics approaches would facilitate resolving what phylotypes are primary contributors to active metabolic pathways and producers of metabolites, as well as how alpha-diversity reductions impact these activities and outputs.

Finally, the inferred microbiome metabolic capacity analyses described here relied upon bacterial proteome databases that lack information for poorly annotated parts of bacterial genomes. Mean proteome annotation coverage across the bacterial tree of life ranges from ~52% to 79%, depending on whether total protein or functional domains are being used (46). As such, functional novelty that may be polyphyletic or unique to specific lineages remains unaccounted for in these analyses. If the latter, then the loss of these lineages would result in a metabolic capacity reduction. More complete annotations of gut bacterial genomes, especially those from under sampled or recently identified lineages, would enable a better estimation of the degree to which functional redundancy maintains metabolic capacity in complex microbiomes.

### Conclusion

Functional redundancy is expected in phylotype-rich microbiomes, which have been hypothesized as being positively selected for because they may buffer against perturbations that could reduce host fitness (i.e., insurance hypothesis [47, 48]). How ecosystem productivity is maintained or not following biodiversity losses is a long-standing topic of inquiry, with ecology and conservation biology being trailblazing disciplines (49). Genome-informed metabolic capacity prediction conservatively captures ecosystem productivity *potentiality*, and it can be observed in experimental microbiomes before and after phylotype loss. We explored how perturbations of a phylotype-rich animal gut microbiome could be taxonomically variable but appear to remain functionally stable. Model insects that have manipulable microbiomes that are phylotype- and function-rich, like the American cockroach, are ideal for uncovering how microbiome compositionality impacts host growth, survival, and fitness. Inferred microbiome metabolic capacity assessments can be easily integrated into 16S rDNA amplicon-based phylotype surveys of microbiomes and they can provide important insights into how phylotype fluctuations may be impacting microbiome services.

## Data Availability

The sequences generated from this experiment were submitted to the NCBI GenBank under the following accession numbers: MF601345–MF601448. All data analyses can be found in the GitHub repository: https://github.com/kbcross/cockroach_frass_microbiome_fr.

## SUPPLEMENTAL MATERIAL

Supplemental figures and legends aem.02368-24-s0001.pdf Figures S1, S2 and S3A-B, and all supplemental legends.

Supplemental tables aem.02368-24-s0002.xlsx Tables S1 to S7.

ASM does not own the copyrights to Supplemental Material that may be linked to, or accessed through, an article. The authors have granted ASM a non-exclusive, world-wide license to publish the Supplemental Material files. Please contact the corresponding author directly for reuse.

## References

[1] Moran NA, Ochman H, Hammer TJ. 2019. Evolutionary and ecological consequences of gut microbial communities. Annu Rev Ecol Evol Syst 50:451–475. doi:10.1146/annurev-ecolsys-110617-062453 32733173 PMC7392196

[2] Ma’ayan A. 2017. Complex systems biology. J R Soc Interface 14:20170391. doi:10.1098/rsif.2017.0391 28931638 PMC5636275

[3] Weisburg WG, Barns SM, Pelletier DA, Lane DJ. 1991. 16S ribosomal DNA amplification for phylogenetic study. J Bacteriol 173:697–703. doi:10.1128/jb.173.2.697-703.1991 1987160 PMC207061

[4] Douglas GM, Maffei VJ, Zaneveld JR, Yurgel SN, Brown JR, Taylor CM, Huttenhower C, Langille MGI. 2020. PICRUSt2 for prediction of metagenome functions. Nat Biotechnol 38:685–688. doi:10.1038/s41587-020-0548-6 32483366 PMC7365738

[5] Boon E, Meehan CJ, Whidden C, Wong D-J, Langille MGI, Beiko RG. 2014. Interactions in the microbiome: communities of organisms and communities of genes. FEMS Microbiol Rev 38:90–118. doi:10.1111/1574-6976.12035 23909933 PMC4298764

[6] Lozupone CA, Stombaugh JI, Gordon JI, Jansson JK, Knight R. 2012. Diversity, stability and resilience of the human gut microbiota. Nature 489:220–230. doi:10.1038/nature11550 22972295 PMC3577372

[7] Louca S, Polz MF, Mazel F, Albright MBN, Huber JA, O’Connor MI, Ackermann M, Hahn AS, Srivastava DS, Crowe SA, Doebeli M, Parfrey LW. 2018. Function and functional redundancy in microbial systems. Nat Ecol Evol 2:936–943. doi:10.1038/s41559-018-0519-1 29662222

[8] Louca S, Jacques SMS, Pires APF, Leal JS, Srivastava DS, Parfrey LW, Farjalla VF, Doebeli M. 2017. High taxonomic variability despite stable functional structure across microbial communities. Nat Ecol Evol 1:0015. doi:10.1038/s41559-016-0015 28812567

[9] Martiny JBH, Jones SE, Lennon JT, Martiny AC. 2015. Microbiomes in light of traits: a phylogenetic perspective. Science 350:aac9323. doi:10.1126/science.aac9323 26542581

[10] Edwards C-C, McConnel G, Ramos D, Gurrola-Mares Y, Dhondiram Arole K, Green MJ, Cañas-Carrell JE, Brelsfoard CL. 2023. Microplastic ingestion perturbs the microbiome of Aedes albopictus (Diptera: Culicidae) and Aedes aegypti. J Med Entomol 60:884–898. doi:10.1093/jme/tjad097 37478409

[11] Moya A, Ferrer M. 2016. Functional redundancy-induced stability of gut microbiota subjected to disturbance. Trends Microbiol 24:402–413. doi:10.1016/j.tim.2016.02.002 26996765

[12] Pérez-Cobas AE, Maiques E, Angelova A, Carrasco P, Moya A, Latorre A. 2015. Diet shapes the gut microbiota of the omnivorous cockroach Blattella germanica. FEMS Microbiol Ecol 91:fiv022. doi:10.1093/femsec/fiv022 25764470

[13] Mu C, Yang Y, Luo Z, Guan L, Zhu W. 2016. The colonic microbiome and epithelial transcriptome are altered in rats fed a high-protein diet compared with a normal-protein diet. J Nutr 146:474–483. doi:10.3945/jn.115.223990 26843585

[14] Tinker KA, Ottesen EA. 2016. The core gut microbiome of the American cockroach, Periplaneta americana, is stable and resilient to dietary Shifts. Appl Environ Microbiol 82:6603–6610. doi:10.1128/AEM.01837-16 27590811 PMC5086554

[15] Tyson GW, Chapman J, Hugenholtz P, Allen EE, Ram RJ, Richardson PM, Solovyev VV, Rubin EM, Rokhsar DS, Banfield JF. 2004. Community structure and metabolism through reconstruction of microbial genomes from the environment. Nature 428:37–43. doi:10.1038/nature02340 14961025

[16] Caporaso JG, Lauber CL, Walters WA, Berg-Lyons D, Huntley J, Fierer N, Owens SM, Betley J, Fraser L, Bauer M, Gormley N, Gilbert JA, Smith G, Knight R. 2012. Ultra-high-throughput microbial community analysis on the Illumina HiSeq and MiSeq platforms. ISME J 6:1621–1624. doi:10.1038/ismej.2012.8 22402401 PMC3400413

[17] Degnan PH, Ochman H. 2012. Illumina-based analysis of microbial community diversity. ISME J 6:183–194. doi:10.1038/ismej.2011.74 21677692 PMC3246231

[18] Edgar RC. 2010. Search and clustering orders of magnitude faster than BLAST. Bioinformatics 26:2460–2461. doi:10.1093/bioinformatics/btq461 20709691

[19] Prosser JI. 2012. Ecosystem processes and interactions in a morass of diversity. FEMS Microbiol Ecol 81:507–519. doi:10.1111/j.1574-6941.2012.01435.x 22715974

[20] Pruesse E, Quast C, Knittel K, Fuchs BM, Ludwig W, Peplies J, Glöckner FO. 2007. SILVA: a comprehensive online resource for quality checked and aligned ribosomal RNA sequence data compatible with ARB. Nucleic Acids Res 35:7188–7196. doi:10.1093/nar/gkm864 17947321 PMC2175337

[21] Quast C, Pruesse E, Yilmaz P, Gerken J, Schweer T, Yarza P, Peplies J, Glöckner FO. 2013. The SILVA ribosomal RNA gene database project: improved data processing and web-based tools. Nucleic Acids Res 41:D590–6. doi:10.1093/nar/gks1219 23193283 PMC3531112

[22] Yao Z, Cai Z, Ma Q, Bai S, Wang Y, Zhang P, Guo Q, Gu J, Lemaitre B, Zhang H. 2022. Compartmentalized PGRP expression along the dipteran Bactrocera dorsalis gut forms a zone of protection for symbiotic bacteria. Cell Rep 41:111523. doi:10.1016/j.celrep.2022.111523 36260997

[23] Anderson MJ, Crist TO, Chase JM, Vellend M, Inouye BD, Freestone AL, Sanders NJ, Cornell HV, Comita LS, Davies KF, Harrison SP, Kraft NJB, Stegen JC, Swenson NG. 2011. Navigating the multiple meanings of β diversity: a roadmap for the practicing ecologist. Ecol Lett 14:19–28. doi:10.1111/j.1461-0248.2010.01552.x 21070562

[24] Oksanen J, Blanchet FG, Friendly M, Kindt R, Legendre P, Mcglinn D, Minchin PR, O’Hara RB, Simpson GL, Solymos P, Stevens MHH, Szoecs E, Wagner H. 2019. Vegan: community ecology package. R package version 2.4-2. Community ecology package 2.5-6

[25] Simpson G. 2022. Repeated measures PERMANOVA nowhere to find. Available from: https://stats.stackexchange.com/questions/590510/repeated-measures-permanova-nowhere-to-find

[26] Benjamini Y, Hochberg Y. 1995. Controlling the false discovery rate: a practical and powerful approach to multiple testing. J R Stat Soc Ser B 57:289–300. doi:10.1111/j.2517-6161.1995.tb02031.x

[27] Macarthur RH. 1965. Patterns of species diversity. Biol Rev 40:510–533. doi:10.1111/j.1469-185X.1965.tb00815.x

[28] Jost L. 2006. Entropy and diversity. Oikos 113:363–375.

[29] Hill MO. 1973. Diversity and Evenness: a unifying notation and its consequences. Ecology 54:427–432. doi:10.2307/1934352

[30] Roswell M, Dushoff J, Winfree R. 2021. A conceptual guide to measuring species diversity. Oikos 130:321–338. doi:10.1111/oik.07202

[31] Bolker BM, Brooks ME, Clark CJ, Geange SW, Poulsen JR, Stevens MHH, White J-SS. 2009. Generalized linear mixed models: a practical guide for ecology and evolution. Trends Ecol & Evol 24:127–135. doi:10.1016/j.tree.2008.10.008 19185386

[32] Davison AC, Hinkley DV. 1997. Bootstrap methods and their application. Cambridge University press.

[33] Hsieh TC, Ma KH, Chao A. 2016. iNEXT: an R package for rarefaction and extrapolation of species diversity (h ill numbers) . Methods Ecol Evol 7:1451–1456. doi:10.1111/2041-210X.12613

[34] R Core Team. 2018. R: a language and environment for statistical computing. R Foundation for Statistical Computing, Vienna, Austria. Available from: https://www.R-project.org

[35] Bates D, Mächler M, Bolker B, Walker S. 2015. Fitting linear mixed-effects models using Ime 4. J Stat Soft 67. doi:10.18637/jss.v067.i01

[36] Chen I-M, Chu K, Palaniappan K, Ratner A, Huang J, Huntemann M, Hajek P, Ritter SJ, Webb C, Wu D, Varghese NJ, Reddy TBK, Mukherjee S, Ovchinnikova G, Nolan M, Seshadri R, Roux S, Visel A, Woyke T, Eloe-Fadrosh EA, Kyrpides NC, Ivanova NN. 2023. The IMG/M data management and analysis system v.7: content updates and new features. Nucleic Acids Res 51:D723–D732. doi:10.1093/nar/gkac976 36382399 PMC9825475

[37] Barbera P, Kozlov AM, Czech L, Morel B, Darriba D, Flouri T, Stamatakis A. 2019. EPA-ng: massively parallel evolutionary placement of genetic sequences. Syst Biol 68:365–369. doi:10.1093/sysbio/syy054 30165689 PMC6368480

[38] Czech L, Barbera P, Stamatakis A. 2020. Genesis and Gappa: processing, analyzing and visualizing phylogenetic (placement) data. Bioinformatics 36:3263–3265. doi:10.1093/bioinformatics/btaa070 32016344 PMC7214027

[39] Louca S, Doebeli M. 2018. Efficient comparative phylogenetics on large trees. Bioinformatics 34:1053–1055. doi:10.1093/bioinformatics/btx701 29091997

[40] Ye Y, Doak TG. 2009. A parsimony approach to biological pathway reconstruction/inference for genomes and metagenomes. PLoS Comput Biol 5:e1000465. doi:10.1371/journal.pcbi.1000465 19680427 PMC2714467

[41] Caspi R, Billington R, Keseler IM, Kothari A, Krummenacker M, Midford PE, Ong WK, Paley S, Subhraveti P, Karp PD. 2020. The MetaCyc database of metabolic pathways and enzymes - a 2019 update. Nucleic Acids Res 48:D445–D453. doi:10.1093/nar/gkz862 31586394 PMC6943030

[42] Turnbaugh PJ, Ley RE, Mahowald MA, Magrini V, Mardis ER, Gordon JI. 2006. An obesity-associated gut microbiome with increased capacity for energy harvest. Nature 444:1027–1031. doi:10.1038/nature05414 17183312

[43] Lützhøft DO, Bækgård C, Wimborne E, Straarup EM, Pedersen K-M, Swann JR, Pedersen HD, Kristensen K, Morgills L, Nielsen DS, Hansen AK, Bracken MK, Cirera S, Christoffersen BØ. 2024. High fat diet is associated with gut microbiota dysbiosis and decreased gut microbial derived metabolites related to metabolic health in young Göttingen Minipigs. PLoS ONE 19:e0298602. doi:10.1371/journal.pone.0298602 38427692 PMC10906878

[44] Raspa F, Chessa S, Bergero D, Sacchi P, Ferrocino I, Cocolin L, Corvaglia MR, Moretti R, Cavallini D, Valle E. 2024. Microbiota characterization throughout the digestive tract of horses fed a high-fiber vs. a high-starch diet. Front Vet Sci 11:1386135. doi:10.3389/fvets.2024.1386135 38807937 PMC11130486

[45] Plichta DR, Juncker AS, Bertalan M, Rettedal E, Gautier L, Varela E, Manichanh C, Fouqueray C, Levenez F, Nielsen T, Doré J, Machado AMD, de Evgrafov MCR, Hansen T, Jørgensen T, Bork P, Guarner F, Pedersen O, Metagenomics of the Human Intestinal Tract (MetaHIT) Consortium, Sommer MOA, Ehrlich SD, Sicheritz-Pontén T, Brunak S, Nielsen HB. 2016. Transcriptional interactions suggest niche segregation among microorganisms in the human gut. Nat Microbiol 1:16152. doi:10.1038/nmicrobiol.2016.152 27564131

[46] Lobb B, Tremblay B-M, Moreno-Hagelsieb G, Doxey AC. 2020. An assessment of genome annotation coverage across the bacterial tree of life. Microb Genom 6:000341. doi:10.1099/mgen.0.000341 PMC7200070 32124724

[47] Ley RE, Peterson DA, Gordon JI. 2006. Ecological and evolutionary forces shaping microbial diversity in the human intestine. Cell 124:837–848. doi:10.1016/j.cell.2006.02.017 16497592

[48] Yachi S, Loreau M. 1999. Biodiversity and ecosystem productivity in a fluctuating environment: the insurance hypothesis. Proc Natl Acad Sci U S A 96:1463–1468. doi:10.1073/pnas.96.4.1463 9990046 PMC15485

[49] McNaughton SJ. 1977. Diversity and stability of ecological communities: a comment on the role of empiricism in ecology. Am Nat 111:515–525. doi:10.1086/283181

