# Supplementary material for: Microbiome metabolic capacity is buffered against phylotype losses by functional redundancy
Source: Appl Environ Microbiol. 2025 Jan 30;91(2):e02368-24. doi: 10.1128/aem.02368-24 (PMC11837509; doi:10.1128/aem.02368-24)
Supplement: Supplemental figures and legends — Figures S1, S2 and S3A-B, and all supplemental legends. [file aem.02368-24-s0001.pdf]

Microbiome metabolic capacity is buffered against perturbation-induced phylotype losses by functional redundancies

Kayla Cross, Noelle Beckman, Benjamin Jahnes, Zakee Sabree

SUPPLEMENTAL MATERIALS

Cover Page – Page 1

Supplemental Figure and Table Legends – Pages 2-4

Figure S1 – Page 5

Figure S2 – Page 6

Figure S3A – Page 7

Figure S3B – Page 8

Supplemental Tables S1-S7 found in "Supplemental Tables.xlsx"

Figure S1. Fecal bacterial abundance was not significantly reduced after feeding on unbalanced diets. 16S rDNA amplicon copies per ug DNA were determined using a standard curve and asterisks indicate significant differences between unbalanced diet samples and week 1 balanced diet samples (Wilcoxon test, $p < 0.05$). Each dot in the boxplot represents a biological sample. Three technological replicates were performed for all biological replicates.

Figure S2. Bacterial pathway redundancy despite phylotype variation. The relative abundance of functional profiles of MetaCyc pathway classes remain consistent across time and between diets, while taxonomic composition varies. Average relative abundance values (y axis) across time (x axis) for phylotypes at family level **(A)** and PICRUSt2-inferred MetaCyc pathways collapsed at into a higher order class (Level 2) **(B)**. The legend highlights the most prevalent phylotypes and MetaCyc classes but are not comprehensive. * Indicate top ten phylotypes (families) and MetaCyc classes**.**

Fig S3. Phylotype loss has minimal impact on metabolic capacity due to pathway redundancy across phylotypes. Pathway presence (non-zero values) and absence (zero values) in cellulose-enrichment (A) and protein-enrichment (B) diet treatments were compared to balanced diet treatment. Pathways remained detectable across the treatment weeks (tan), were undetectable across treatment weeks (white), detectable only at the end of the treatment (red), or undetectable at the end of the treatment

(black). Comparisons for metabolic pathway detectability were between microbiome samples of age-matched insects (i.e., balanced diet weeks 3– and 4 vs. cellulose-enriched diet weeks 3– and 4; balanced diet weeks 7– and 8 vs. protein-enriched diet weeks 7– and 8). The *y*-axis depicts bacterial and archaeal families ordered by phylum. The *x*-axis is MetaCyc pathway names grouped by a broad MetaCyc category (top) and ordered within the category from most shared to least shared pathways (bottom). Pathways that are a member of a variant class by MetaCyc are indicated by an asterisks, followed by the variant class name.

Table S1. Number of samples per treatment per week that passed quality-filtering. Detailed are the number of samples for which there were >5,000 high-quality amplicons.

Table S2. Diet alters microbiome composition. PERMANOVA analysis indicated that diet (p-value < 0.05) accounted for the differences in community profiles between a control (nutritionally-balanced) and treatment (protein-enriched and cellulose-enriched) diets. Permutations were restricted within individuals.

Table S3. Dietary shifts explain differences in dispersions.
P values were adjusted using the Benjamini-Hochberg False Discovery Rate (FDR).

Table S4. Protein- and cellulose-enriched diets lead to a decrease in bacterial families. Bacterial family diversity within diets (balanced, protein-enriched, and cellulose-enriched) have a downward trend of bacterial family loss demonstrated by negative

coefficient estimate and confidence interval overlapping zero. Intercept: mean of nutrient balanced diet in week one; Coef. Est: Coefficient estimates that are the differences between the nutrient balanced mean and the other two imbalanced diets. The coefficient estimate is the change in phylotype diversity with one unit increase in week for the nutrient balanced diet; Week: slope of nutrient balanced; Protein x Week and Cellulose x Week: differences in slope between nutrient balanced and imbalanced diets; SD: standard deviation. The diversity between balanced and the two unbalance diets are similar if the confidence interval (2.5% - 97.5%) for "Protein x Week" or "Cellulose x Week" overlaps zero.

Table S5. Total Pathways. All MetaCyc pathways detected across all treatments. Values adjacent to MetaCyc pathway names reflect the number of samples in each treatment that the pathway was detected.

Table S6. Obligately-Shared Pathways. All MetaCyc pathways detected across all treatments that required gene products from two or more phylotypes to complete the metabolic pathway. Values adjacent to MetaCyc pathway names reflect the number of samples in each treatment that the pathway was detected.

Table S7. Per-Taxon Pathways. All MetaCyc pathways detected across all treatments for which a single phylotype encoded all of the gene products needed to complete the metabolic pathway. Values adjacent to MetaCyc pathway names reflect the number of samples in each treatment that the pathway was detected.

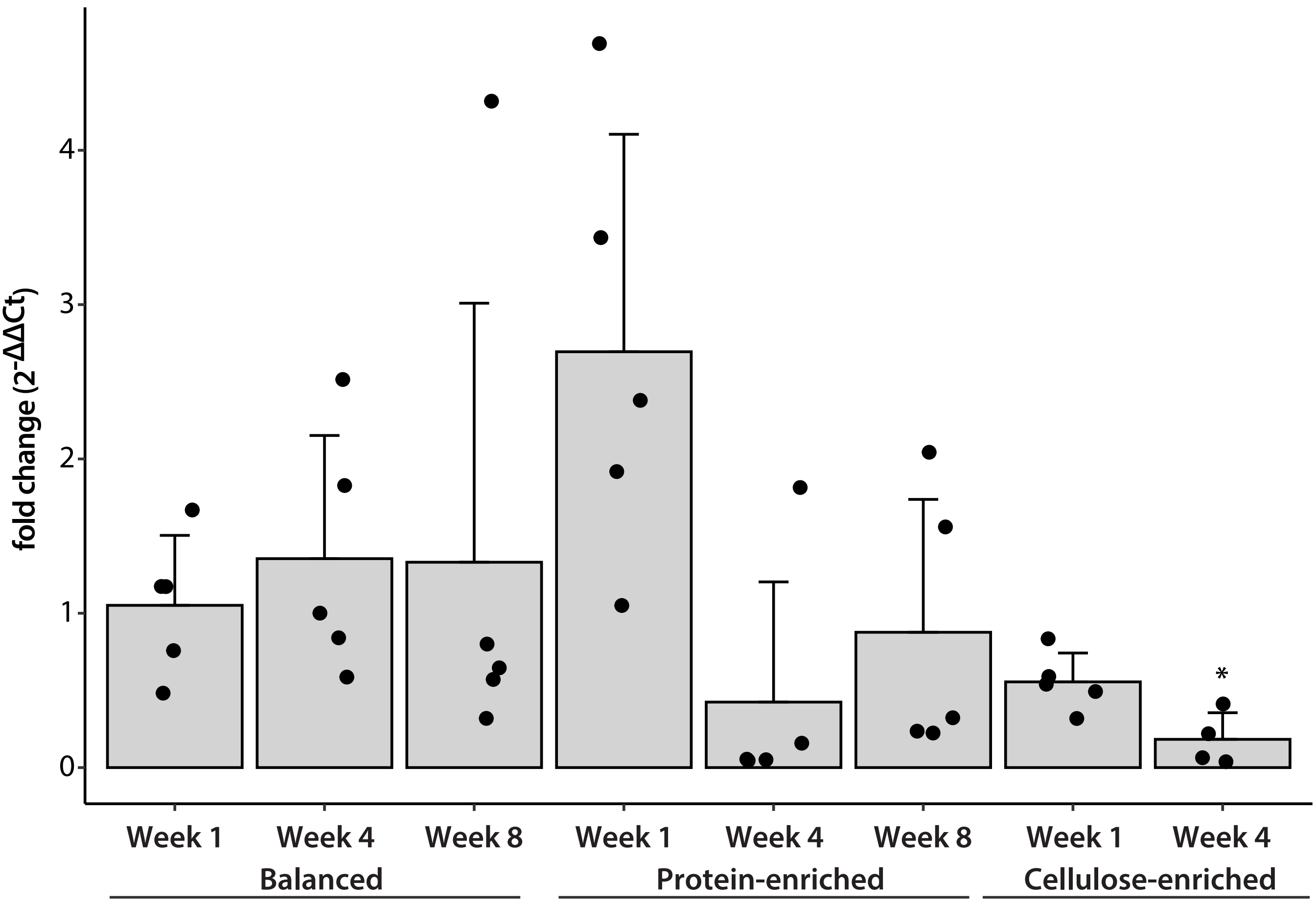
fold change ($2^{-\Delta\Delta Ct}$)
0
1
2
3
4
*
Week 1
Week 4
Week 8
Week 1
Week 4
Week 8
Week 1
Week 4
Balanced
Protein-enriched
Cellulose-enriched

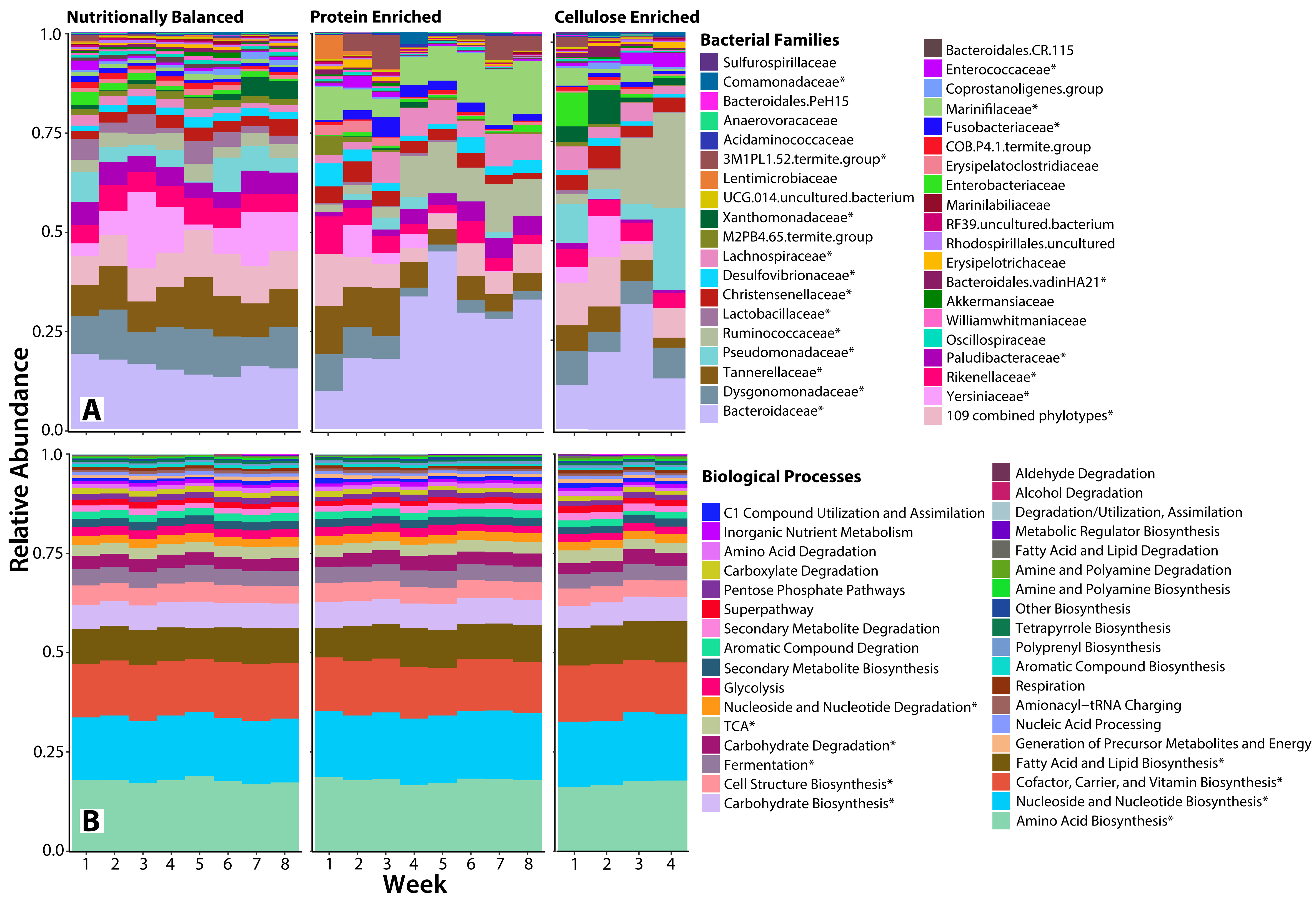

Nutritionally Balanced
Protein Enriched
Cellulose Enriched
Relative Abundance
Week
A
B
0.0
0.25
0.5
0.75
1.0
1
2
3
4
5
6
7
8
Bacterial Families
Sulfurospirillaceae
Comamonadaceae*
Bacteroidales.PeH15
Anaerovoraceae
Acidaminococcaceae
3M1PL1.52.termite.group*
Lentimicrobiaceae
UCG.014.uncultured.bacterium
Xanthomonadaceae*
M2PB4.65.termite.group
Lachnospiraceae*
Desulfovibrionaceae*
Christensenellaceae*
Lactobacillaceae*
Ruminococcaceae*
Pseudomonadaceae*
Tannerellaceae*
Dysgonomonadaceae*
Bacteroidaceae*
Bacteroidales.CR.115
Enterococcaceae*
Coprostanoligenes.group
Marinifilaceae*
Fusobacteriaceae*
COB.P4.1.termite.group
Erysipelatoclostridiaceae
Enterobacteriaceae
Marinilabiliaceae
RF39.uncultured.bacterium
Rhodospirillales.uncultured
Erysipelotrichaceae
Bacteroidales.vadinHA21*
Akkermansiaceae
Williamwhitmaniaceae
Oscillospiraceae
Paludibacteraceae*
Rikenellaceae*
Yersiniaceae*
109 combined phylotypes*
Biological Processes
C1 Compound Utilization and Assimilation
Inorganic Nutrient Metabolism
Amino Acid Degradation
Carboxylate Degradation
Pentose Phosphate Pathways
Superpathway
Secondary Metabolite Degradation
Aromatic Compound Degration
Secondary Metabolite Biosynthesis
Glycolysis
Nucleoside and Nucleotide Degradation*
TCA*
Carbohydrate Degradation*
Fermentation*
Cell Structure Biosynthesis*
Carbohydrate Biosynthesis*
Aldehyde Degradation
Alcohol Degradation
Degradation/Utilization, Assimilation
Metabolic Regulator Biosynthesis
Fatty Acid and Lipid Degradation
Amine and Polyamine Degradation
Amine and Polyamine Biosynthesis
Other Biosynthesis
Tetrapyrrole Biosynthesis
Polyprenyl Biosynthesis
Aromatic Compound Biosynthesis
Respiration
Amionacyl–tRNA Charging
Nucleic Acid Processing
Generation of Precursor Metabolites and Energy
Fatty Acid and Lipid Biosynthesis*
Cofactor, Carrier, and Vitamin Biosynthesis*
Nucleoside and Nucleotide Biosynthesis*
Amino Acid Biosynthesis*

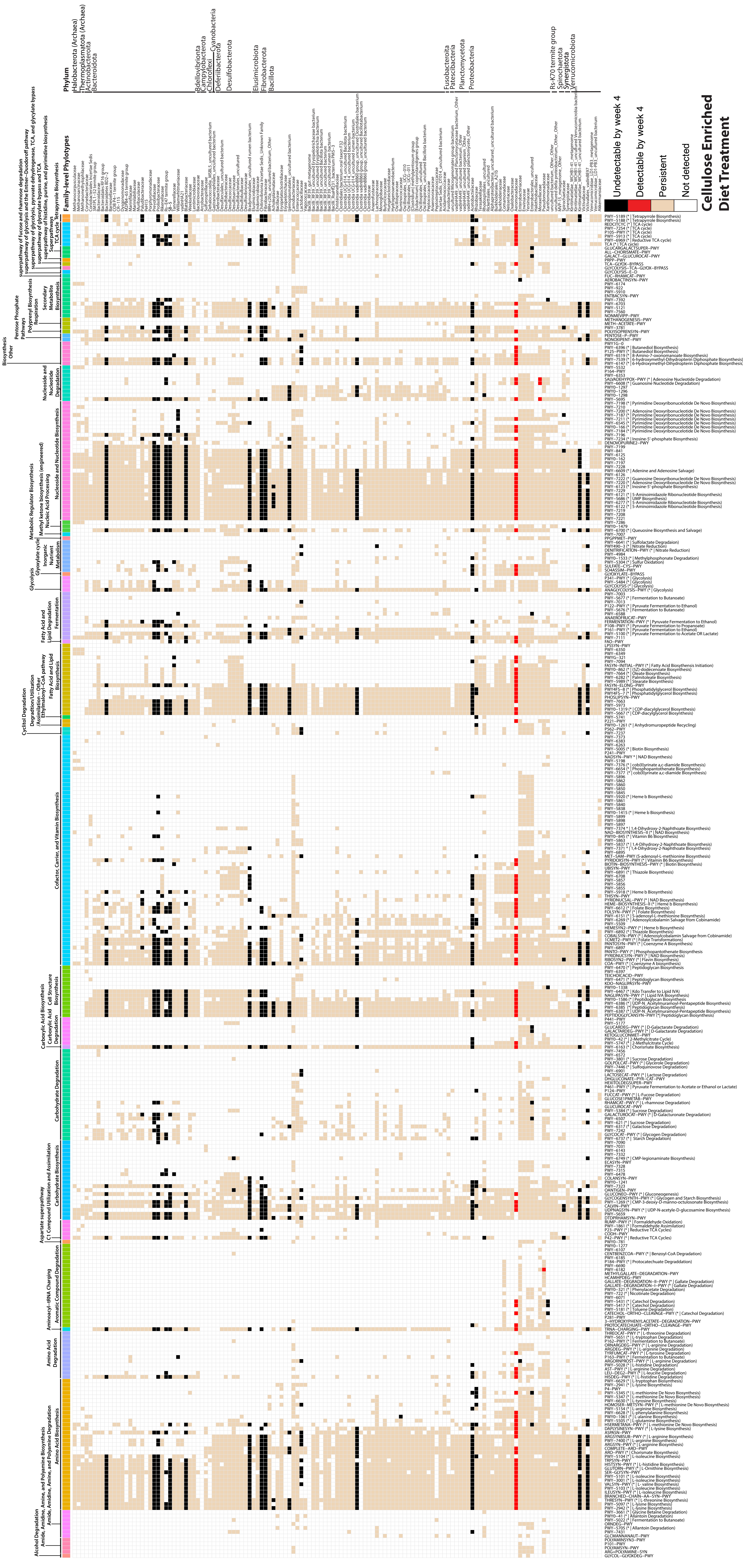
Cellulose Enriched Diet Treatment
Undetectable by week 4
Detectable by week 4
Persistent
Not detected
Phylum
Family-level Phylotypes

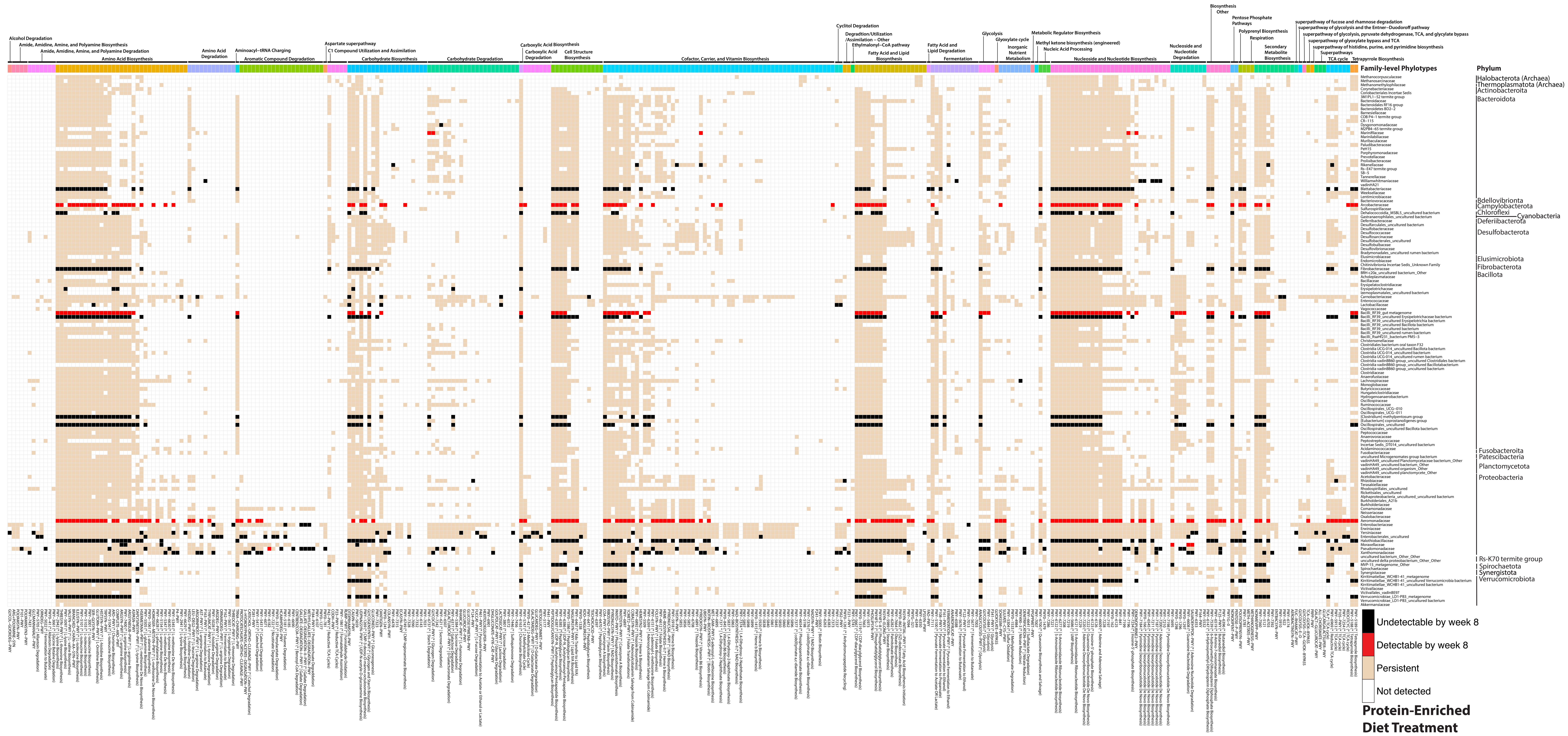

Alcohol Degradation
Amide, Amidine, Amine, and Polyamine Biosynthesis
Amide, Amidine, Amine, and Polyamine Degradation
Amino Acid Biosynthesis
Amino Acid Degradation
Aminoacyl–tRNA Charging
Aromatic Compound Degradation
Aspartate superpathway
C1 Compound Utilization and Assimilation
Carbohydrate Biosynthesis
Carbohydrate Degradation
Carboxylic Acid Biosynthesis
Carboxylic Acid Degradation
Cell Structure Biosynthesis
Cofactor, Carrier, and Vitamin Biosynthesis
Cyclitol Degradation
Degradation/Utilization /Assimilation – Other
Ethylmalonyl–CoA pathway
Fatty Acid and Lipid Biosynthesis
Fatty Acid and Lipid Degradation
Fermentation
Glycolysis
Glyoxylate cycle
Inorganic Nutrient Metabolism
Metabolic Regulator Biosynthesis
Methyl ketone biosynthesis (engineered)
Nucleic Acid Processing
Nucleoside and Nucleotide Biosynthesis
Nucleoside and Nucleotide Degradation
Biosynthesis Other
Pentose Phosphate Pathways
Polyprenyl Biosynthesis
Respiration
Secondary Metabolite Biosynthesis
superpathway of fucose and rhamnose degradation
superpathway of glycolysis and the Entner–Duodoroff pathway
superpathway of glycolysis, pyruvate dehydrogenase, TCA, and glycolate bypass
superpathway of glyoxylate bypass and TCA
superpathway of histidine, purine, and pyrimidine biosynthesis
Superpathways
TCA cycle
Tetrapyrrole Biosynthesis
Family-level Phylotypes
Phylum
Halobacterota (Archaea)
Thermoplasmatota (Archaea)
Actinobacteriota
Bacteroidota
Bdellovibrionta
Campylobacterota
Chloroflexi
Cyanobacteria
Deferribacterota
Desulfobacterota
Elusimicrobiota
Fibrobacterota
Bacillota
Fusobacteriota
Patescibacteria
Planctomycetota
Proteobacteria
Rs-K70 termite group
Spirochaetota
Synergistota
Verrucomicrobiota
Undetectable by week 8
Detectable by week 8
Persistent
Not detected
Protein-Enriched Diet Treatment
